# Supplementary material for: Characterization of Two Novel Bacillus thuringiensis Cry8 Toxins Reveal Differential Specificity of Protoxins or Activated Toxins against Chrysomeloidea Coleopteran Superfamily
Source: Toxins (Basel). 2020 Oct 5;12(10):642. doi: 10.3390/toxins12100642 (PMC7599620; doi:10.3390/toxins12100642)
Supplement: Supplementary file 1 [file toxins-12-00642-s001.pdf]

Supplementary Materials: Characterization of Two Novel *Bacillus thuringiensis* Cry8 Toxins Reveal Differential Specificity of Protoxins or Activated Toxins Against Chrysomeloidea Coleopteran Superfamily

Changlong Shu, Guixin Yan, Shizhi Huang, Yongxin Geng, Mario Soberón, Alejandra Bravo, Lili Geng and Jie Zhang

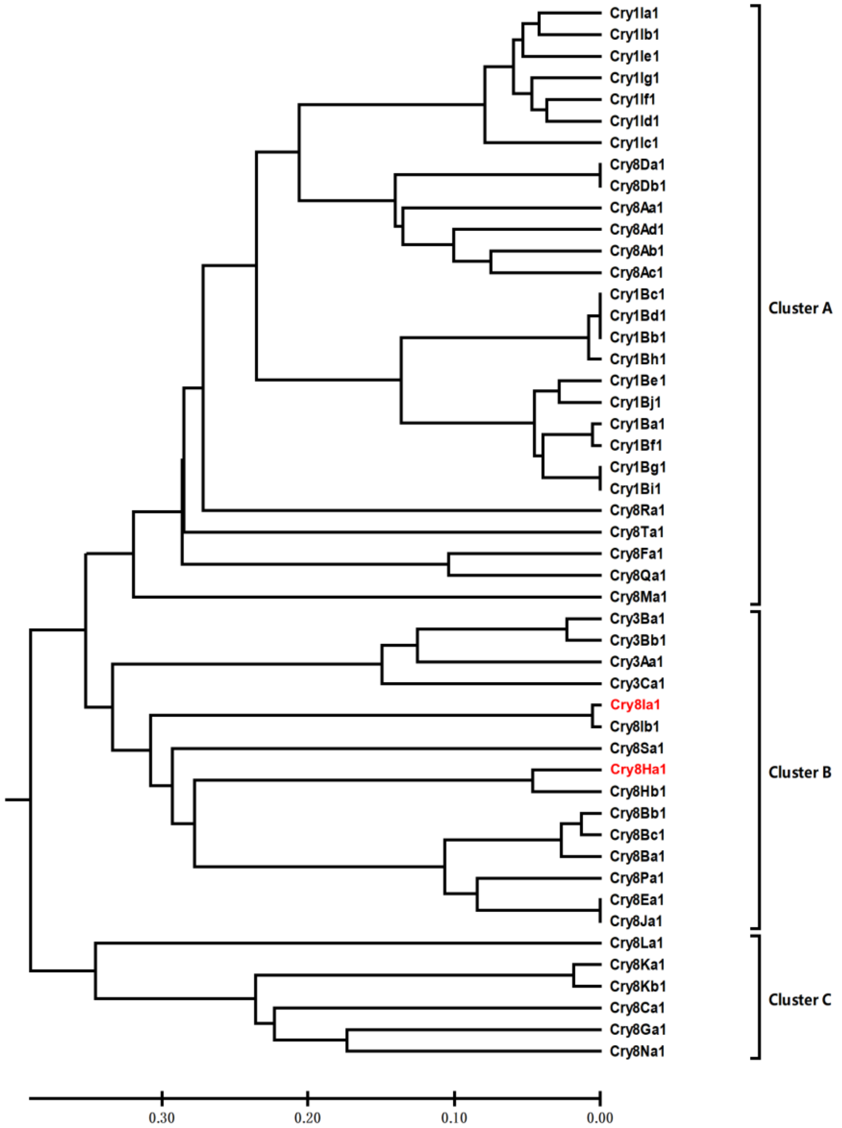

Figure S1. UPGMA phylogenetic analysis of the amino acid sequences from domain I of Cry proteins. The distances were computed using the Poisson correction method and are in the units of the number of amino acid substitutions per site. These analyses involved 49 amino acid sequences. All ambiguous positions were removed for each sequence pair (pairwise deletion option). Evolutionary analyses were conducted in MEGA X. The Cry8Ha1 and Cry8Ia1 proteins were labeled in red letters.

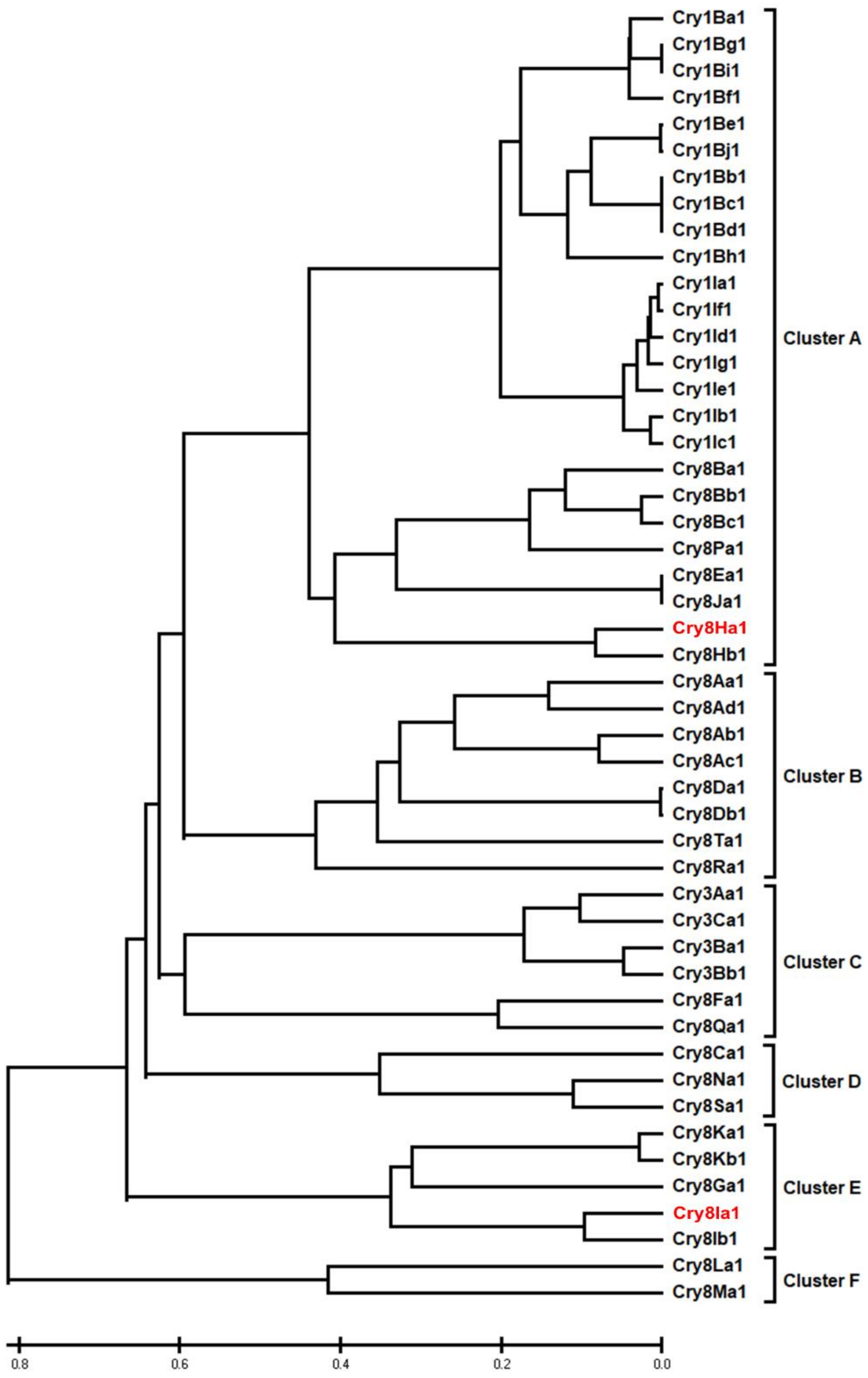

Figure S2. UPGMA phylogenetic analysis of the amino acid sequences from domain II of Cry proteins. The distances were computed using the Poisson correction method and are in the units of the number of amino acid substitutions per site. These analyses involved 49 amino acid sequences. All ambiguous positions were removed for each sequence pair (pairwise deletion option). Evolutionary analyses were conducted in MEGA X. The Cry8Ha1 and Cry8Ia1 proteins were labeled in red letters.

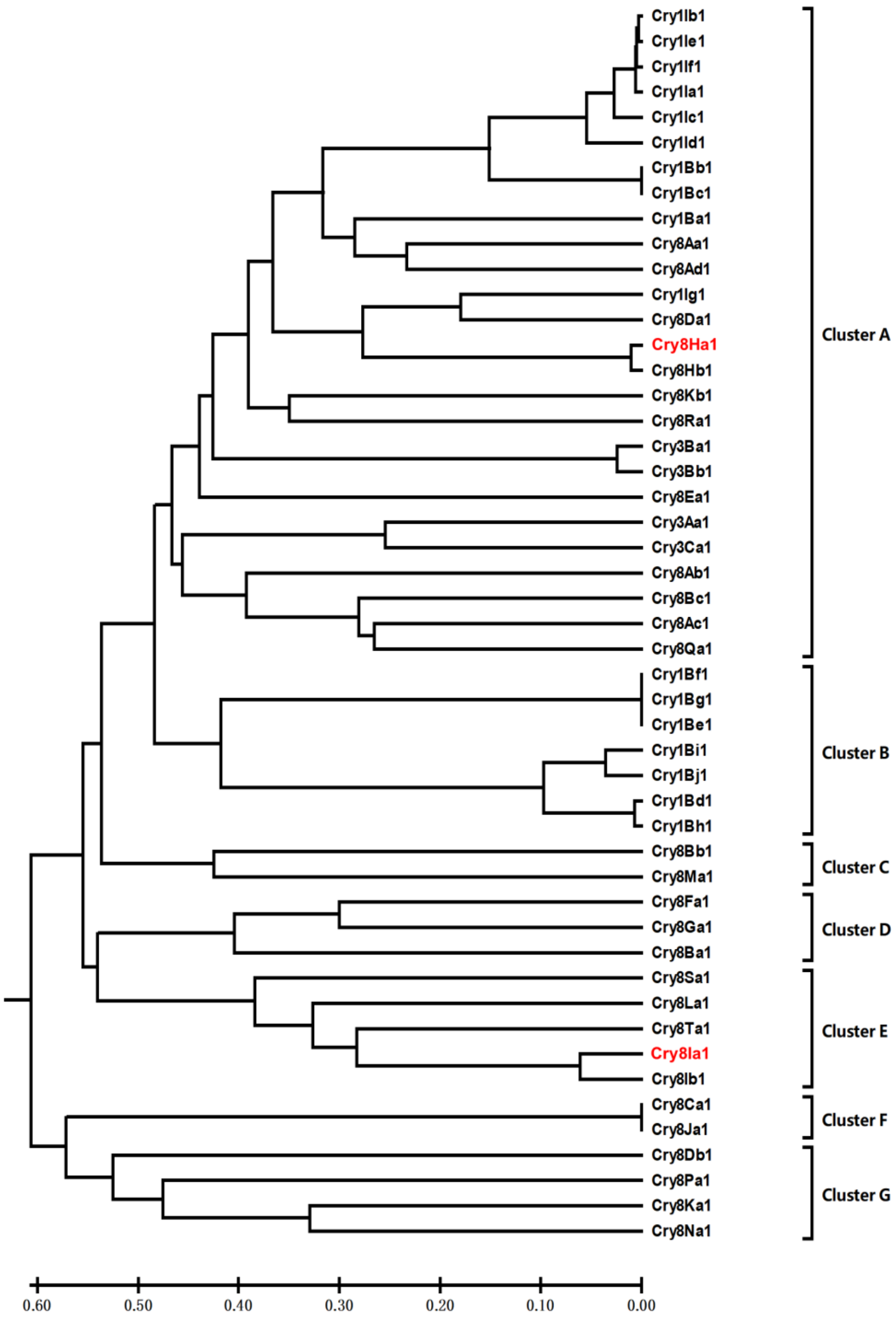

Figure S3. UPGMA phylogenetic trees of the amino acid sequences from domain III of Cry proteins. The distances were computed using the Poisson correction method and are in the units of the number of amino acid substitutions per site. These analyses involved 49 amino acid sequences. All ambiguous positions were removed for each sequence pair (pairwise deletion option). Evolutionary analyses were conducted in MEGA X. The Cry8Ha1 and Cry8Ia1 proteins were labeled in red letters.
